# Early Breathing in Very Preterm Infants during Deferred Cord Clamping (DCC) Is Related to Gestational Age and Is Not Independently Associated with Important Neonatal Outcomes: A 5-Year Cohort Study

**DOI:** 10.3390/children11030347

**Published:** 2024-03-14

**Authors:** Michael P. Meyer, Elizabeth Nevill

**Affiliations:** 1 Neonatal Unit, KidzFirst, Middlemore Hospital, Auckland 2025, New Zealand; elizabeth.nevill@cmdhb.org.nz; 2 Department of Paediatrics Child and Youth Health, University of Auckland, Auckland 1010, New Zealand

**Keywords:** preterm infant, deferred cord clamping, resuscitation, early breathing, birth transition, neonatal outcomes

## Abstract

Deferred cord clamping (DCC) has been associated with reduced mortality in preterm infants, and a period of at least 30 s has been recommended before clamping. However, preterm infants assessed as being in need of resuscitation have often had earlier cord clamping. In this study, we aimed to compare neonatal outcomes for preterm infants undergoing DCC who established early breathing movements compared to those who were not breathing. After a 5 yr recruitment period, we recently completed the ABC study, in which preterm infants <31 weeks undergoing 50 s of DCC who were not breathing by 15 s of age were randomised into two groups: one received intermittent positive pressure ventilation (IPPV) and the other was a standard group, which received no breathing support. The outcomes in the two groups were similar, and for the present analysis, the groups were combined. Infants in the ABC study were compared with the cohort excluded from the original ABC study because they were breathing by 15 s (called the Breathing Before Clamping or BBC group). There were significant differences in demographics between the ABC and BBC groups. Spontaneous preterm labour was more common in the BBC group, and these infants were more likely to be delivered vaginally. Gestational age and birth weight were significantly higher in the BBC group (*p* < 0.01). Soon after birth, Apgar scores were significantly higher in the BBC group, with a lower base deficit on first obtained blood gas, and a smaller proportion were intubated in the delivery room. Fewer BBC infants were hypothermic (<36.5 °C) on admission. Multivariate regression analysis indicated whether infants were breathing or not at 15 s of age was linked predominantly to gestation. Important neonatal outcomes and a composite of these outcomes (mortality, severe intraventricular haemorrhage, bronchopulmonary dysplasia) were not significantly different between the ABC and BBC groups (odds ratio for the composite outcome was 1.77 CI 0.84–3.76 corrected for gestation). For very preterm infants undergoing DCC, important neonatal outcomes were related to gestational age and not independently associated with early breathing. There was a small group (7% of total) who were deemed compromised at birth and did not undergo DCC. These infants had significantly worse neonatal outcomes.

## 1. Introduction

Deferred cord clamping (DCC) of at least 30 s has been recommended for very preterm infants, although, more recently, a minimum of 60 s has been proposed [1,2]. There have been a number of systematic reviews comparing DCC with immediate cord clamping (ICC). A recent one, on which the ILCOR (International Liaison Committee on Resuscitation) recommendation of at least 30 s of DCC was based, included only infants <34 weeks gestation [1]. There were almost 3000 infants (in total) from 16 randomised studies. The main finding was an improved survival (of 2%). Following this, a recent meta-analysis of individual participant data (IPD) and a network meta-analysis (NMA) were performed [3,4]. This approach confirmed, with a high certainty of evidence, the reduced death rate before discharge in infants receiving DCC. Other findings were a reduction in red blood cell transfusions and reduced need for inotropes following delivery. The NMA also suggested that longer periods of DCC (>60 s) might led to greater reductions in mortality. However, a review of the recent studies indicated that 30% or more did not actually have DCC because of maternal/perinatal events such as placental abruption or because the infants were assessed as compromised [5].

It is hard to define which babies might be in need of or would benefit from breathing support during DCC [6,7]. Assessment may be aided by parameters such as heart rate, which, for a number of reasons, is difficult to accurately assess over 30 to 60 s while DCC is taking place [8,9]. Likewise, the infant’s colour may not be a helpful sign as oxygen saturations are low immediately after birth [10]. Irregular breathing or apnoea while the infant is still being perfused via the cord during DCC has uncertain significance. There are a number of studies investigating this [11], and during the recently completed New Zealand (NZ) ABC (Assisted Breathing Before Clamping) study, we found no benefit to providing intermittent positive pressure ventilation (IPPV) in a group of <31-week-old infants undergoing 50 s of DCC [12].

Briefly, in the NZ ABC study, preterm infants who were deemed not to be breathing at 15 s after birth were randomised to either standard care or to receive IPPV [8]. During standard care, infants were gently stimulated by rubbing the back and torso. After 50 s, the cord was clamped. The intervention group received IPPV with a T piece resuscitator and blended gas. Apart from small changes in the early acid base parameters, there were no significant differences in neonatal outcomes between the two groups [12].

In the current study, we wished to compare important neonatal outcomes in the cohort of infants excluded from the ABC study because they had established regular early breathing by 15 s of age. We called this cohort the BBC group (Breathing Before Clamping). In addition, we aimed to further describe the cohort excluded from DCC because of their compromised condition at birth. In future, this group may benefit from more advanced resuscitation with an intact cord.

## 2. Materials and Methods

**Figure 1:**
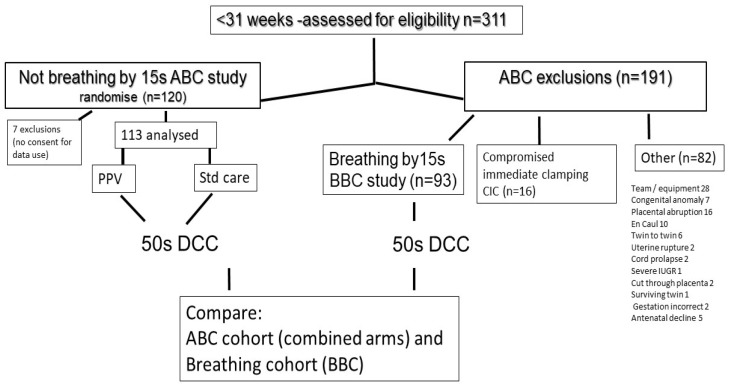
Modified CONSORT diagram for group allocation to ABC (Assisted Breathing Before Clamping), BBC (Breathing Before Clamping), and CIC (Compromised Immediate Clamping) groups. PPV—positive pressure ventilation; Std—standard care; DCC—deferred cord clamping; IUGR—intrauterine growth retardation.

During the NZ ABC study, over a 5-year period at a single centre, there were 311 infants < 31 weeks assessed. Of these, 120 were randomised to the NZ ABC study, and 113 were analysed (7 parents declined use of the data from their infants). There were 198 exclusions (see Figure 1). In total, 54 infants were excluded for maternal or perinatal reasons, e.g., abruption and en caul delivery, and another 28 were excluded for other reasons, e.g., equipment not ready or declined antenatal consent. There were 93 infants excluded because they were breathing regularly by 15 s (Breathing Before Clamping; BBC). This group also received 50 s of DCC (as was our routine hospital practice). These BBC infants were compared with the ABC group in relation to demographics and neonatal outcomes. For this purpose, since there were no clinically significant differences between the ABC infants related to the provision of IPPV or standard care, the 2 arms of the ABC study were combined into a single group of infants not breathing by 15 s. An additional 16 infants were deemed in need of immediate resuscitation because, in addition to being apnoeic, they had decreased activity and tone. These infants were excluded from both the ABC and BBC groups and referred to as the CIC (Compromised and Immediate Clamping) group. Of 222 infants whose breathing was assessed by the research team (311-82), these 16 infants comprised 7% of the total.

Neonatal data were routinely collected for the Australian and New Zealand Neonatal Network (ANZNN) repository for all infants <32 weeks gestational age. For the purposes of this study, routinely collected ANZNN data for the BBC and CIC groups were used for the comparisons with the ABC group. Complete ANZNN network definitions are reported elsewhere [13]. Briefly, gestational age was based on the first day of the last menstrual period, BPD was defined as a requirement for oxygen or respiratory support at 36 weeks post-menstrual age (PMA) and for babies <28 weeks a physiologic definition was used, severe IVH was regarded as IVH grade 3 or 4 on cranial ultrasound, and necrotising enterocolitis (NEC) was modified Bell stage 2 or more or diagnosis at postmortem or surgery. For comparisons, a composite neonatal outcome defined as death, severe IVH, or BPD during the neonatal admission was used.

Statistics: Medians and interquartile ranges were reported for data not known to be normally distributed, and nonparametric tests, e.g., the Mann–Whitney U test, were utilised for these data. Chi square and Fishers exact test were used for the calculation of proportions and odds ratios. Logistic regression was used to account for confounding variables and beta co-efficients with odds ratios reported (SPSS 26). For the regression analyses, all the demographic variables in Table 1 were entered. For regression, gestational age was used rather than birth weight, and antenatal intrauterine growth restriction (IUGR) was entered separately.

## 3. Results

The participant flow for the ABC study was described previously [12], and for the whole cohort, it is shown in Figure 1. Maternal and infant demographics are shown in Table 1.

Ethics: Both the ABC and BBC studies were approved by local and national ethics review committees.

There were significant differences between the ABC and BBC groups. Spontaneous preterm labour was a less common presentation for the mothers of ABC infants, whilst pre-eclampsia was more common. Antenatal magnesium sulphate (given < 30 weeks) was more often given to women whose infants were in the ABC group. The receipt of antenatal steroids (both complete and incomplete courses) was not significantly different. ABC infants were more likely to be delivered by caesarean section (CS). Median gestation was one week lower in the ABC group, and median birth weight was almost 200 g lighter. All the BBC infants had DCC; there were three who underwent DCC for 45 s rather than 50 s, but there was no apparent reason for this slightly shorter time (Table 2). In the ABC group, 93% of infants underwent 50 s of DCC. Early neonatal outcomes (Apgar scores, first blood gas results, delivery room intubation, and admission temperature within desired range) were all significantly worse in the ABC group. Although raw data showed a trend toward a worse composite outcome of death, BPD, and severe IVH in the ABC group, once corrected for confounders (especially gestational age), there was no significant difference (OR 1.77 CI 0.84–3.76 Table 2 and Table 3). Significantly more infants received a blood transfusion in the ABC group (OR 2.57 CI 1.26–5.24). However, using multivariate analysis, gestational age was the only significant variable, and group assignment was not significant (OR 1.18 95% CI 0.38–3.65).

Overall, for infants that could be assessed at 15 s who underwent DCC, 120 (ABC group) of 213 (ABC + BBC groups) or 56% had not established breathing by 15 s. Using logistic regression analysis to determine the factors predictive of being in the ABC or BBC group (i.e., breathing regularly by 15 s), gestational age and vaginal birth were the only variables of significance. When infants were grouped into those above or below 28 weeks, mode of delivery and gestation showed a significant association with breathing by 15 s. Similarly, only gestational age was predictive of the composite outcome (group assignment, i.e., ABC or BBC cohort, was not a significant variable; Table 4). Being in the ABC cohort was, however, predictive of a 1 min Apgar score of <4 even after correcting for confounders (OR 6.30 CI 1.64–24.22).

**Figure 2:**
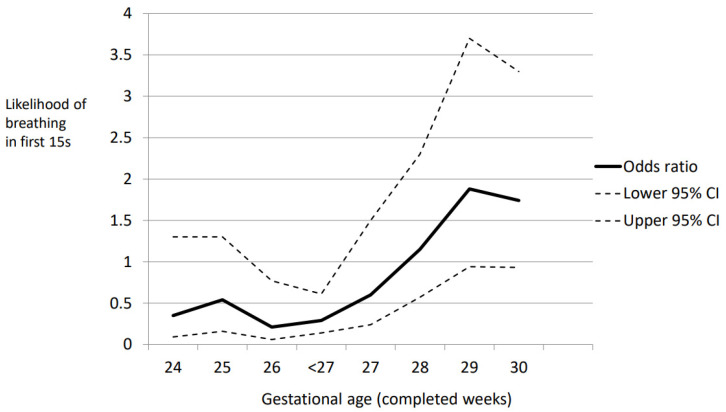
Likelihood of breathing by 15 s expressed as odds ratios and confidence intervals at different gestational ages for infants in ABC (Assisted Breathing Before Clamping) and BBC (Breathing Before Clamping) groups. Gestational age < 27 weeks includes all infants below this gestation, while the individual odds ratios for each week < 27 weeks are also shown.

Odds ratios (and 95% CI) of breathing or not at 15 s for each week of gestation were calculated (Figure 2). From the graph, an OR of one was present between 27 and 28 weeks gestation.

Compared to the ABC group, the group of 16 infants deemed to be too compromised at birth to receive DCC and who therefore underwent immediate clamping (CIC) had lower 1 and 5 min Apgar scores and a greater base deficit (Supplementary Table S1). In total, 15 of the 16 CIC infants had at least one component of the composite outcome. However, when examining factors which may be predictive of being in the CIC group, we found that antenatal and birth demographics were not significantly different than the ABC group (Supplementary Table S2).

## 4. Discussion

In this cohort study, infants excluded from the ABC study because they were judged to be breathing regularly by 15 s (BBC group) were of a significantly higher birth weight and gestational age and, when corrected for the latter, did not have significantly different neonatal outcomes in respect of blood transfusion or the composite outcome from the ABC cohort. The early establishment of respiration was related only to gestational age, with other study variables not significantly affecting this association. Although there were antenatal differences between groups, these were no longer significant after correcting for gestation.

Calculating the likelihood of breathing by 15 s or not for each week of gestation, an odds ratio of 1 was apparent for infants between 27 and 28 weeks PMA. However, because confidence intervals were wide, the prediction of early breathing based only on gestation is unlikely to be reliable. In a study of preterm infants < 32 weeks undergoing DCC, age in seconds at which breathing was established was noted [11]. Approximately 40 percent had established breathing by 15–20 s [14], which is similar to our finding. In a study of more mature infants (median 37 weeks) who were undergoing DCC, the median time to first cry was 11 s in the non-compromised group [15].

The standard care group of the ABC cohort continued to have 50 s of DCC regardless of their breathing effort, and at the end of the 50 s, only 36% had established regular breathing movements. The results of the ABC study and the BBC cohort indicate that during (time-based) 50 s of DCC, lack of early breathing was not by itself an indication to cease DCC. Similarly, in the study of Katheria et al., lack of breathing was not used as an indication to stop DCC, but in this study, only 10% were not breathing by 60 s [11]. This difference to the NZ ABC study likely reflects the different selection criteria for study entry, but the conclusions of both were similar.

Apart from breathing, other assessments of the infant’s condition during DCC were limited to tone and activity, and for ABC babies, this was judged to be appropriate. It was difficult to consistently assess heart rate during the ABC study because there was one neonatal attendee providing plastic wrap for thermal control, positioning the infant and providing either gentle stimulation or PPV. Without more intensive monitoring (e.g., heart rate, oxygen saturations), we chose a relatively short period of DCC. It is currently uncertain whether longer periods of DCC would be safe and more beneficial than 50 s in the group who have not established regular breathing efforts. However, time to clamping could reasonably be extended to 60 s to bring it in line with proposed new ILCOR guidelines [2]. On the basis of this observational cohort study, neonatal outcomes were not independently improved in the group of preterm infants that were breathing. This suggests that adopting a physiological basis to DCC and not clamping the cord until breathing is established (Physiological-Based Cord Clamping or PBCC) may not improve neonatal outcomes. A similar finding was noted in a study which recruited more mature infants in need of resuscitation [15].

A recent network meta-analysis using individual participant data and a time-based approach to DCC indicated that periods longer than 60 s were associated with greater reductions in mortality compared to shorter periods. However, three of the five studies included in this NMA were carried out in more mature infants (>32 weeks) in relatively resource-poor settings, and it is uncertain if these data can be extrapolated to other populations and more preterm infants [4]. Several studies are investigating longer times before cord clamping and providing breathing support with an intact cord or following a cord milking approach. Providing resuscitation with an intact cord until breathing and gas exchange are achieved has been carried out in a multicentre study in the Netherlands. A comparison between a standard group receiving time-based DCC (60 s) and a group receiving PBCC has been performed, and the peer-reviewed results are awaited [16]. Two other multicentre studies have been completed, but they have not yet been published, and the results of these studies will help inform practice regarding this important question [17,18].

The infants excluded from the ABC study because they were assessed as being compromised (not breathing, poor tone, and activity by 15 s of age) had a significantly worse condition in the first few minutes after birth and worse composite outcomes. With more intensive monitoring or support, it may be possible to continue to resuscitate these infants while still attached to the cord and aim for a more physiological basis to determine when to clamp their cords. However, in deciding beforehand which infants might require this approach, we could not identify them from the collected variables, and overall, the CIC group comprised only 7% of the total population of preterm infants assessed for DCC.

The strengths of the current study include that the full cohort of preterm infants <31 weeks were described in relation to DCC and whether or not they established early breathing. For the ABC study, careful attention was paid to early respiratory assessment. Those not breathing could have been deemed to be “in need of resuscitation” and excluded from some studies. Neonatal outcomes from the cohort were available from either the ABC study or from the BBC audit (ANZNN database). While the single-centre nature of this study ensured uniformity of care practices, and that the 5 yr observational period may increase generalisability, there were also limitations. There was a relatively small sample size, and some confidence intervals were wide. Breathing movements, as we recorded, might not be associated with lung inflation as we did not measure gas exchange in the BBC cohort. Monitoring heart rate during DCC was generally not achievable at our centre. Neonatal outcomes may not reflect longer-term follow up data.

## 5. Conclusions

Infants excluded from the ABC study because they were breathing at 15 s had a more mature gestational age, but when corrected for this, they had similar neonatal outcomes to the ABC cohort. This indicates that within the parameters of the study (50 s DCC), early breathing on its own was not a useful indicator with regard to deciding when to clamp the cord.

## Figures and Tables

**Table 1:** Demographics of Assisted Breathing Before Clamping (ABC) group and Breathing Before Clamping (BBC) groups. Results shown as number (%) or median (IQR).

Characteristic	ABC Cohort (113)	BBC Cohort (93)	*p* Value or OR (95%CI)
Preterm labour n (%)	60 (53)	77 (82)	0.37 (0.20–0.68)
Pre-eclampsia n (%)	33 (29)	10 (11)	3.46 (1.60–7.49)
Antepartum bleeding n (%)	38 (34)	30 (32)	1.08 (0.60–1.94)
Antenatal IUGR n (%)	25 (22)	12 (13)	1.94 (0.92–4.12)
Antenatal steroid complete n (%)	82 (74)	67 (72)	0.91 (0.49–1.69)
Antenatal magnesium sulphate (any) n (%)	94 (83)	60 (64)	0.37 (0.19–0.71)
Multiple birth n (%)	21 (19)	27 (29)	0.56 (0.29–1.07)
Vaginal delivery n (%)	31 (27)	49 (52)	0.35 (0.19–0.62)
Male n (%)	59 (52)	53 (56)	0.79 (0.45–1.36)
Gestational age (weeks) median (IQR)	28 (26–29)	29 (28–30)	<0.01
Birth weight (g) median (IQR)	1120 (847–1335)	1315(1107–1502)	<0.01

**Table 2:** Neonatal outcomes of Assisted Breathing Before Clamping (ABC) group and Breathing Before Clamping (BBC) groups. Results shown as n (%) or median (IQR).

Outcome	ABC Cohort (113)	BBC Cohort (93)	*p* or OR
DCC 50 sec n (%)	105 (93)	90 (97)	0.35
Worst base excess mmol/L	−4 (−7 to −1)	−3 (−5 to 0)	0.03
First lactate mmol/L	3.1 (1.9–6.1)	2.1 (1.5–3.0)	0.01
Apgar 1 min	6 (3–7)	7 (6–9)	0.01
Apgar 5 min	8 (7–9)	9 (8–9)	0.01
Apgar 1 min <4 (n%)	28 (26)	3 (3)	9.88 (2.90–33.71)
Intubation in DR (n%)	23 (20)	4 (4)	5.75 (1.91–17.30)
Adm temp <36.5 °C (n%)	26 (25)	9 (10)	2.82 (1.25–6.38)
Surfactant given (n%)	58 (55)	43 (46)	1.20 (0.70–2.09)
Infection (n%)	31 (27)	22 (23)	1.22 (0.65–2.30)
Blood transfusion (n%)	33 (29)	13 (14)	2.57 (1.26–5.24)
BPD (n%)	35 (31)	18 (19)	1.92 (1.00–3.68)
Severe IVH (n%)	11 (10)	3 (3)	3.34 (0.91–12.36)
Death (n%)	9 (8.5)	2 (2)	3.94 (0.83–18.70)
Composite outcome (n%)	47 (45)	27 (28)	1.74 (0.97–3.12)

Adm temp—admission temperature, BPD—bronchopulmonary dysplasia, DCC—deferred cord clamping, DR—delivery room, IVH—intraventricular haemorrhage.

**Table 3:** Variables associated with a worse composite outcome: logistic regression analysis of ABC (Assisted Breathing Before Clamping) and BBC (Breathing Before Clamping) cohorts.

	Significance	Odds Ratio	95% CI	
			Lower	Upper
Preterm labour	0.551	0.706	0.224	2.221
APH	0.325	1.452	0.691	3.051
Antenatal IUGR	0.149	0.440	0.144	1.343
Multiple birth	0.936	0.967	0.430	2.177
Sex	0.164	1.597	0.826	3.088
Gestation	0.000	0.691	0.576	0.829
Vaginal birth	0.553	1.292	0.553	3.017
PET	0.715	1.262	0.362	4.396
Antenatal steroid	0.928	1.035	0.489	2.192
ABC group	0.134	1.774	0.838	3.756
Constant	0.001	8604.655		

APH—antepartum haemorrhage, IUGR—intrauterine growth restriction, PET—pre-eclampsia, ABC group—Assisted Breathing Before Clamping.

**Table 4:** Variables associated with early breathing: results of logistic regression analysis for ABC (Assisted Breathing Before Clamping) and BBC infants.

	Significance	Odds Ratio	95% CI	
			Lower	Upper
Preterm labour	0.840	0.898	0.316	2.548
APH	1.000	1.000	0.489	2.044
Antenatal IUGR	0.808	0.878	0.308	2.505
Multiple birth	0.116	0.547	0.258	1.160
Sex	0.522	1.233	0.649	2.341
Gestation	0.000	1.608	1.334	1.939
Vaginal birth	0.002	3.792	1.632	8.811
PET	0.071	2.883	0.914	9.090
Antenatal steroid	0.770	0.896	0.428	1.873
Constant	0.000	0.000		

APH—antepartum haemorrhage, IUGR—intrauterine growth restriction, PET—pre-eclampsia, ABC group—Assisted Breathing Before Clamping.

## Data Availability

As data for BBC infants were obtained from de-identified information, data will not be available.

## Supplementary Materials

The following supporting information can be downloaded at: https://www.mdpi.com/article/10.3390/children11030347/s1, Table S1: Demographics of Assisted Breathing Before Clamping (ABC) group and Compromised with Immediate Clamping (CIC) groups and Table S2: Neonatal Outcomes of Assisted Breathing Before Clamping (ABC) group and Compromised with Immediate Clamping (CIC) groups.
