# Supplementary material for: Early Breathing in Very Preterm Infants during Deferred Cord Clamping (DCC) Is Related to Gestational Age and Is Not Independently Associated with Important Neonatal Outcomes: A 5-Year Cohort Study
Source: Children (Basel). 2024 Mar 14;11(3):347. doi: 10.3390/children11030347 (PMC10968951; doi:10.3390/children11030347)
Supplement: Supplementary file 1 [file children-11-00347-s001.zip › children-2821486-supplementary.pdf]

Supplementary Table S1. Demographics of Assisted Breathing Before Clamping (ABC) group and Compromised with Immediate Clamping (CIC) groups.

Characteristic	ABC cohort (113)	CIC cohort (16)	p value or OR (95%CI)
Preterm labour n (%)	60 (53)	5 (31)	2.49 (0.81-7.63)
Pre eclampsia n (%)	33 (29)	4 (25)	1.24 (0.37-4.12)
Antepartum bleeding n (%)	38 (34)	7 (44)	0.65 (0.22-1.88)
Antenatal IUGR n (%)	25 (22)	1 (6)	4.93 (0.62-39.26)
Antenatal steroid complete n (%)	82 (74)	9 (56)	2.06 (0.71-6.00)
Multiple birth n (%)	21 (19)	1 (6)	3.42 (0.43-27.38)
Vaginal delivery n (%)	31 (27)	6 (38)	0.63 (0.21-1.88)
Male n (%)	59 (52)	11 (69)	0.50 (0.16-1.52)
Gestational age (weeks) median (IQR)	28 (26-29)	26 (24-30)	0.26
Birth weight (g) median (IQR)	1120 (847-1335)	948 (774-1291)	0.22

IUGR – intrauterine growth restriction.

Supplementary Table S2. Neonatal Outcomes of Assisted Breathing Before Clamping (ABC) group and Compromised with Immediate Clamping (CIC) groups. Results are number (%) or median (IQR)

Outcome	ABC cohort (113)	CIC cohort (16)	P or OR
DCC 50 sec	105 (93)	0	<0.001
Worst base excess mmol/l	-4 (-7 to -1)	-7 (-13 to -4)	0.03
First lactate mmol/l	3.1 (1.9-6.1)	6.0 (3.0-10.0)	0.60
Apgar 1 min	6 (3-7)	3 (1-4)	<0.001
Apgar 5 min	8 (7-9)	5 (3-8)	0.003
Apgar 1 min <4	28 (26)	7 (44)	4.52 (1.53-13.37)
Intubation in DR	23 (20)	8 (50)	1.96 (0.61-6.29)
Adm temp <36.5°C	26 (25)	6 (38)	0.50 (0.17-1.50)
Surfactant given	58 (55)	10 (63)	0.63 (0.22-1.86)
Infection	31 (27)	7 (44)	2.12 (0.71-6.32)
BPD	35 (31)	10 (63)	5.38 (1.57-18.42)
Severe IVH	11 (10)	3 (25)*	3.06 (0.71-13.31)
Death	9 (8.5)	4 (25)	3.03 (0.83-9.03)
Composite outcome	47 (45)	15 (94)	23.7 (3.00-187.6)

Adm temp – admission temperature, BPD – bronchopulmonary dysplasia, IVH – intraventricular haemorrhage. *Denominator 12
